# Rationally Programming Nanomaterials with DNA for Biomedical Applications

**DOI:** 10.1002/advs.202003775

**Published:** 2021-02-24

**Authors:** Liangcan He, Jing Mu, Oleg Gang, Xiaoyuan Chen

**Affiliations:** ^1^ Yong Loo Lin School of Medicine and Faculty of Engineering National University of Singapore Singapore 117597 Singapore; ^2^ Institute of Precision Medicine Peking University Shenzhen Hospital Shenzhen 518036 China; ^3^ Department of Chemical Engineering and Department of Applied Physics and Applied Mathematics Columbia University New York NY 10027 USA; ^4^ Center for Functional Nanomaterials Brookhaven National Laboratory Upton NY 11973 USA

**Keywords:** biomedical applications, DNA conjugation, DNA nanostructures, DNA origami, dynamic clusters

## Abstract

DNA is not only a carrier of genetic information, but also a versatile structural tool for the engineering and self‐assembling of nanostructures. In this regard, the DNA template has dramatically enhanced the scalability, programmability, and functionality of the self‐assembled DNA nanostructures. These capabilities provide opportunities for a wide range of biomedical applications in biosensing, bioimaging, drug delivery, and disease therapy. In this review, the importance and advantages of DNA for programming and fabricating of DNA nanostructures are first highlighted. The recent progress in design and construction of DNA nanostructures are then summarized, including DNA conjugated nanoparticle systems, DNA‐based clusters and extended organizations, and DNA origami‐templated assemblies. An overview on biomedical applications of the self‐assembled DNA nanostructures is provided. Finally, the conclusion and perspectives on the self‐assembled DNA nanostructures are presented.

## Graphical abstract

DNA is not only a carrier of genetic information, but also a versatile structural tool for the engineering and self‐assembling of nanostructures. In this regard, the DNA template has dramatically enhanced the scalability, programmability, and functionality of the self‐assembled DNA nanostructures. These capabilities provide opportunities for the biomedical applications in biosensing, bioimaging, drug delivery, and disease therapy.

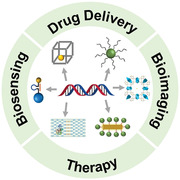

## 1 Introduction

Deoxyribonucleic acid (DNA) is a kind of biomolecules composed of nucleobases, which string the sequence to form a chain of polynucleotide.^[^ ^1^ ^]^ In particular, there are four basic nucleobases (including adenine (A), thymine (T), cytosine (C), and guanine (G)) in DNA. The number and sequence of basic nucleobases in the polynucleotide chain determine the encoding information and structural function of the DNA.^[^ ^1^ ^]^ Based on the Watson–Crick pairing interactions, helical duplex or more complex structures can be formed from the basic nucleobases (A pairs with T, C pairs with G). Naturally, it is easy to hold together multiple building units (such as colloid nanoparticles) via the specific pairing interactions.

In 1982, DNA was first proposed as a structural material by Seeman,^[^ ^2^ ^]^ and later utilized as a programmable bond and linker for ordered architecture construction.^[^ ^3^, ^4^, ^5^, ^6^, ^7^, ^8^, ^9^ ^]^ To date, 2D or 3D periodic and aperiodic superstructures have been made based on the DNA base pairs interactions.^[^ ^10^, ^11^, ^12^, ^13^, ^14^ ^]^ Typically, the precise DNA nanostructures can be designed and realized at nanoscale reflected by the length of 0.33 nm bp^−1^.^[^ ^1^, ^15^ ^]^ In addition, the specific insertion and deletion of DNA base pairs enable the possibility of controlling the stress and flexibility of DNA nanostructures to construct complex morphologies with curves.^[^ ^16^ ^]^ Recently, the emerging DNA origami technology offers new opportunities for the fabrication of multifunctional DNA nanostructures at spatially precise levels. To this end, DNA origami templates are widely used for precise conjugation of functional nanoparticles or biomolecules, resulting in new optic and theranostic nanodevices.

**Scheme 1:**
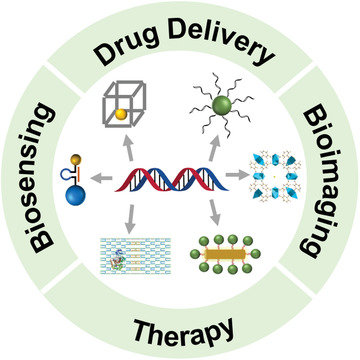
Schematic illustration of DNA programmed nanomaterials for biomedical applications.

In this review, we first point out the importance of using DNA template (including the DNA linker and DNA origami) for the fabrication and application of DNA nanostructures. Second, we discuss diverse types of self‐assembled DNA nanostructures. Third, we introduce the biomedical applications of the DNA assembled nanoplatforms (**Scheme** **1**). Last, we prospect the existing challenges and point out the possible opportunities in the development of DNA nanostructures.

## 2 Why DNA Conjugation?

In the past decade, great efforts have been made in the rational design of functional metal nanostructures with well‐defined sizes and designed morphologies due to the unique physical and chemical properties and broad applications in the field of sensing, catalysis, and cancer theranostics.^[^ ^17^, ^18^, ^19^, ^20^ ^]^ Therefore, various top‐down and bottom‐up strategies have been proposed for the construction of diverse metal nanostructures.^[^ ^21^, ^22^, ^23^ ^]^ In particular, the DNA‐based bottom‐up nanofabrication has been widely explored recently, mainly due to the precisely controllable feature of DNA nanostructures.^[^ ^24^ ^]^ Different from other biomolecules (such as peptide and protein), the DNA is a kind of biopolymer with ordered base pairing as the building units. The characters of physiological stability, tunable size, and programmable architecture make the DNA not only act as a powerful tool for nanoassembly, but also endow the generated nanoassemblies with more emerging functionalities. Given the above advantages, the DNA nanotechnology is widely used in optics, sensing, and biomedical applications.^[^ ^25^, ^26^ ^]^

1) As a linker, the DNA is very useful in nanomaterials conjugation and construction of DNA hybridization‐mediated complex nanostructures. For individual nanoparticle conjugation, it is still very necessary to develop effective strategies to graft DNA linkers on the surface of nanoparticles. In most current studies, the DNA linkers are mostly conjugated directly or indirectly on the particle surfaces via chemical interactions between the functional group terminated DNA strands and the particles.^[^ ^27^ ^]^ The thiol‐DNA conjugated Au nano particles (Au NPs) are one of the most explored examples, which have been widely used for the construction of complex DNA‐Au superstructures and nanodevices. For self‐assembled DNA nanostructures, it is vital to mediate the interparticle interactions, which can be achieved by controlling the assembly modes of the individual building units.^[^ ^13^, ^28^, ^29^ ^]^ Usually, there are two strategies for the self‐assembly DNA nanostructures: a) direct hybridization, where the DNA conjugated particles directly hybridize with the complementary DNA grafted particles; and b) DNA linker‐mediated hybridization, where two non‐complementary DNA conjugated particles are linked by the third DNA strand which is partially complementary to both DNA strands. By carefully controlling the interparticle interactions, discrete nanocrystals can be organized into spatially predefined nanostructures by using the DNA templates. More importantly, the composition and topology of the DNA nanostructures can also be organized, due to the intrinsic DNA features which are independent of the particle sizes and composition.
2) The DNA conjugation could improve the biocompatibility and stability of the nanostructures, especially for inorganic nanoparticles (such as Au NPs). Recent advances have shown that the DNA nanostructures have demonstrated great promise in the field of sensing, imaging, and cancer theranostics, mainly due to their improved biocompatibility and stability.^[^ ^30^ ^]^ Moreover, designed dynamic DNA structures also allow to switch their assembly state and modify the surfaces in response to the molecular trigger or environmental conditions (the tumor microenvironment (TME) for example).^[^ ^29^, ^31^, ^32^ ^]^
3) The DNA conjugation would also introduce the theranostic functions of DNA (e.g., catalytic nucleic acids (DNAzymes), gene silencing) into the conjugation system.^[^ ^33^, ^34^ ^]^ For example, the DNAzymes based nanoprobes were widely used for metal ions detection.^[^ ^35^, ^36^, ^37^ ^]^ Moreover, the DNAzymes or gene sequences could be delivered in the aid of conjugation with nanoparticles.^[^ ^34^, ^38^ ^]^

## 3 Self‐Assembled DNA Nanostructures

### 3.1 Single Nanoparticle (DNA Conjugated Single Particle) System

DNA is a versatile and powerful ligand for the assembly of nanomaterials by using its programmable and sequence‐specific interactions. In practically, they are explored as “non‐biological” engineering materials for conjugation and self‐assembly of nanoparticle.^[^ ^9^, ^39^, ^40^ ^]^ Recently, great efforts have been done for the modification and self‐assembly of inorganic nanoparticles,^[^ ^27^, ^32^, ^41^, ^42^, ^43^, ^44^, ^45^, ^46^, ^47^, ^48^, ^49^, ^50^, ^51^, ^52^, ^53^ ^]^ inorganic‐organic nanoparticles (metal–organic frameworks (MOFs)),^[^ ^51^, ^54^, ^55^ ^]^ and biomolecules (proteins, enzymes)^[^ ^56^ ^]^ with DNA strands.

#### 3.1.1 Inorganic Nanoparticles

In the 1990s, Mirkin and Alivisatos first described the synthesis of oligonucleotide‐Au conjugates using thiol terminated DNA sequences.^[^ ^3^, ^4^ ^]^ In both of their works, the Au NPs were successfully grafted with thiol‐DNA sequences due to the strong interactions between the Au and thiol. Encouragingly, the DNA‐AuNPs demonstrated enhanced stability in biological solutions, which had great promise in complex nanostructure constructions and applications. In 2006, Mirkin and co‐workers investigated the performances of the DNA functionalized AuNPs (13 nm) as antisense agents.^[^ ^57^ ^]^ Unexpectedly, the AuNP‐antisense oligodeoxyonucleotides (ASNPs) could easily enter into green fluorescent protein‐expressing C166 cells despite their coating with negatively charged DNA sequences. Compared with those unbounded with antisense oligodeoxyonucleotides (ASODNs), the particle bounded with ASODNs degraded much more slowly in the cellular environment, demonstrating its improved biostability. Similarly, Tavallaie et al. developed a highly sensitive sensor for the direct detection of microRNA at very low concentrations (10 am–1 nm, 1 am is 10^−9^ nm) in unprocessed blood samples.^[^ ^58^ ^]^ In their studies, the surface of gold coated magnetic NPs (Au@MNPs) were first modified by thiol‐DNA strands (probe) at one end, and methylene blue redox‐labeling at the other end. The probe DNA strands were complementary to the target miRNA‐21. After exposure of the DNA‐based sensor in the analyte solution for 30 min, the amount of the target analyte could be measured through the electric‐field induced reconfiguration of Au@MNPs network. Furthermore, the sensor showed high sensitively within the region of 1 nm–10 am for direct detection of miRNA‐21 in whole blood samples, demonstrating its promise in clinical applications.

Kelley and co‐workers developed a one‐pot method for the synthesis of luminescent and biofunctionalized ≈6 nm CdTe nanocrystals which could specifically bind DNA, protein, and CCRF‐CEM cells.^[^ ^59^ ^]^ In this synthetic protocol, a chimeric DNA strand was used for CdTe growth and biofunctionalization, one part of which served as ligand phosphorothioate domain (ps) and the other part served as biorecognized phosohate domain (po). The ability of the DNA‐CdTe was confirmed in the detection of complementary DNA or thrombin. The ps‐po DNA strand functionalized CdTe exhibited the highest binding level toward the target agents. Furthermore, the specific binding ability of CdTe nanocrystals was realized in discrimination of cognate cells (CCRF‐CEM and Ramos cells). Similarly, the DNA has also been successfully grafted onto other colloid NPs, such as UCNPs,^[^ ^51^, ^60^ ^]^ TiO_2_,^[^ ^61^, ^62^ ^]^ CuS,^[^ ^63^, ^64^ ^]^ and graphene^[^ ^65^ ^]^ with the aid of specific chemical reactions.

#### 3.1.2 MOFs

MOFs, emerging as a new kind of porous materials, have received great attention in gas adsorption, catalysis, energy, and biomedicine in the past decades, due to their intrinsic characters of high surface areas, adjustable structures, and functionalities.^[^ ^66^, ^67^, ^68^, ^69^ ^]^ In the biomedical applications, the biostability and biocompatibility are the prerequisites. However, most of the current MOFs are not very stable in biological environment. Therefore, it is urgent to develop strategies to improve their biological stability and compatibility. There are two common strategies for the conjugation of MOFs with DNA strands.

The first strategy is to graft DNA on MOFs via a second linker, which bridges the MOFs and the DNA strands. In 2014, Mirkin and co‐workers reported zirconium based UiO‐66‐DNA conjugates.^[^ ^54^ ^]^ In the work, they first synthesized the UiO‐66‐N_3_ NPs by using 2‐azido‐1,4‐benzenedicarboxylic acid and zirconyl chloride octahydrate, where the surface azido groups could be used for the following DNA functionalization via Cu‐free click chemistry. In addition, the DNA strands on the MOF surfaces provided steric and electrostatic barriers to stabilize the MOFs in high dielectric media. Compared to UiO‐66 NPs without DNA conjugation and free DNA sequences, the DNA‐UiO‐66 NPs exhibited about fourfold and sixfold higher cellular uptake, respectively. Later, Willner and co‐workers successfully constructed stimuli‐responsive DNA‐MOFs, in which the amine‐modified i‐motif DNA (cytosine‐rich nucleic acid) strands were conjugated with carboxylate groups on the MOFs.^[^ ^55^ ^]^ Interestingly, the i‐motif DNA functionalized MOFs demonstrated pH‐responsive cargo release properties. At pH 5.5, the loaded rhodamine 6G was blocked by the quadruplex structures, while it could be rapidly released from the pores at pH 7.4 solution resulting from the unfolding of the cytosine‐rich i‐motif DNA strands. Similar strategy could also be applied for the fabrication of ion‐responsive DNA‐MOFs.^[^ ^70^, ^71^, ^72^ ^]^ The controllable “off‐on” or “open‐lock” of the DNA gates paved the way to develop versatile platforms to achieve the controlled drug release.^[^ ^73^, ^74^ ^]^

**Figure 1:**
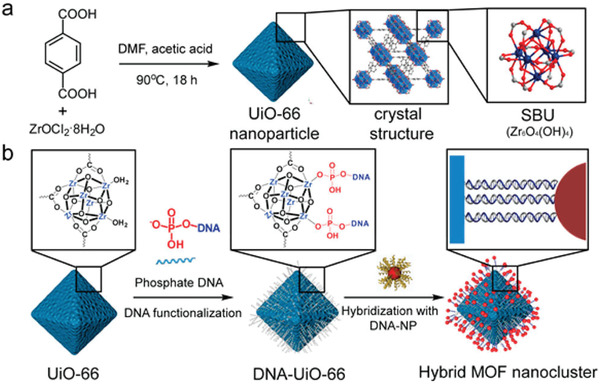
a) Synthesis of UiO‐66 and b) its DNA functionalization via phosphate terminated DNA sequences. Reproduced with permission.^[^ ^76^ ^]^ Copyright 2017, American Chemical Society.

The second strategy is to directly conjugate linker molecule or DNA strands on MOFs via the unsaturated coordination sites (CUS). In particular, a facile way for directly modifying the MOFs with DNA strands via CUS was developed by Mirkin and co‐workers very recently.^[^ ^75^, ^76^ ^]^ In another example reported by Cha and co‐workers, they successfully conjugated the zirconium‐based PCN‐224 MOFs via the zirconium CUS by using *N*_*α*_,*N*_*α*_‐bis(carboxymethyl)‐L‐lysine hydrate as the conjugation bridge.^[^ ^51^ ^]^ It was shown that the PCN‐224 NPs could be easily functionalized with amine groups, followed by conjugation with DBCO‐NHS linker and click chemistry with azide‐DNA strands. The DNA‐PCN‐224 could be programmable assembled with complementary DNA‐modified optical nanoparticles. Instead of using linker molecule, Mirkin and co‐workers directly conjugated a series of CUS MOFs (UiO‐66, UiO‐67‐bpy, UiO‐68‐N_3_, PCN‐222, PCN‐223, PCN‐224, MIL‐101(Fe, Cr, Al)) with phosphate‐terminated DNA strands in one of their recent works (**Figure** **1**).^[^ ^76^ ^]^ By taking advantage of the specific interactions of DNA, various hybrid MOF‐NPs were synthesized, regardless of the size, shape, and composition. To date, the DNA functionalization is limited to a few types of MOFs. Although great progresses have been made on effective modification of MOFs with oligonucleotides, it is still a challenge to develop a versatile strategy to improve their biocompatibility and stability.^[^ ^51^, ^54^, ^55^, ^76^, ^77^, ^78^ ^]^

#### 3.1.3 Biomolecules

Proteins and enzymes are nature's solution to create functional complexity from the limited building blocks.^[^ ^79^ ^]^ In particular, the efficient delivery of functional proteins or enzymes has broad applications in nanomedicine. However, the cellular uptake of proteins or enzymes is impeded by their inherent characters, such as the instability, large size, charged surfaces.^[^ ^80^ ^]^ Alternatively, these issues could be largely solved by the surface coating. For example, peptides were recently shown as a viable molecular coating strategy for stabilizing DNA origami in physiological conditions,^[^ ^30^ ^]^ and structural tunability of peptides architecture can potentially be used for tailoring a DNA uptake. With respect to DNA conjugation, the DNA strands can be grafted on the biomaterials (such as protein and enzyme) through the residual groups on their surfaces, which is similar to the above DNA conjugation strategies on MOFs.^[^ ^81^, ^82^, ^83^, ^84^ ^]^ Encouragingly, Mirkin group reported a chemical strategy for DNA‐mediated cellular delivery of *β*‐galactosidase.^[^ ^85^ ^]^ In their work, there were ≈25 DNA strands conjugated on each *β*‐galactosidase. The covalent attachment of DNA was realized via chemical conjugation between their surface lysine amines and DBCO‐DNA. More importantly, the attachment of DNA strands enhanced its cellular uptake by up to ≈280 fold. This study provides a promising strategy for efficient delivery of proteins. Gang group recently showed that spatially defined DNA frames can be used to organize streptavidin and enzymatic cascades (glucose oxidase (GOx) and horseradish peroxidase (HRP)) into well‐ordered 3D arrays.^[^ ^13^ ^]^ Interestingly, an enzymatic cascade shows an increasing activity, which is promising for establishing rationally fabricated and complexly organized enzymatic scaffolds.

### 3.2 Clusters and Extended Organization (DNA‐Assembled Organizational Structures)

#### 3.2.1 Nanoparticle Clusters

**Figure 2:**
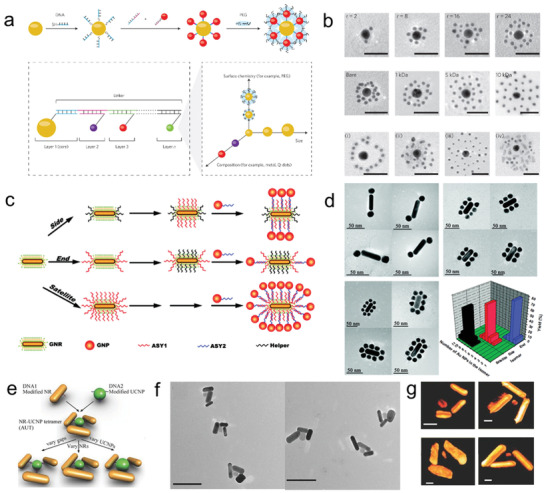
a) Scheme for the self‐assembly of core‐satellite AuNP superstructures and b) the corresponding TEM images. Reproduced with permission.^[^ ^91^ ^]^ Copyright 2014, Springer Nature. c) Regiospecific AuNR‐AuNP assemblies and d) the corresponding TEM images. Reproduced with permission.^[^ ^93^ ^]^ Copyright 2012, American Chemical Society. e) Scheme and f) TEM images of AuNR‐UCNP tetramer assemblies. g) The corresponding cryo‐TEM tomographic reconstructions of NR‐UCNP tetramer assembly. Reproduced with permission.^[^ ^94^ ^]^ Copyright 2016, Wiley‐VCH GmbH.

Due to specific DNA interaction, the DNA‐based assembly provides powerful methods for fabrication of nanoparticle clusters with novel functions.^[^ ^86^, ^87^ ^]^ Gang group demonstrated the stepwise assembly process for creating Janus nanoparticles and formation of dimers and complex clusters from them.^[^ ^88^ ^]^ In the recent studies, the group showed a new approach for creating designed clusters with symmetric and arbitrary arrangement of nanoparticles in space using reprogrammable 3D ball‐like mesh DNA scaffold.^[^ ^89^ ^]^ Recently, Chan and co‐workers constructed a variety of novel Au‐based hybrid nanoassemblies with the aid of DNA template.^[^ ^90^, ^91^, ^92^ ^]^ Different from those in the single particle system, the biological delivery, and elimination properties could be tuned in the DNA assembled core‐satellite (CS) superstructures (**Figure** **2**a).^[^ ^91^ ^]^ In their CS Au NP‐Au NP superstructures, the central core NP was surrounded by one or multiple layers of satellite NPs (Figure 2b). By carefully controlling the DNA conjugation, the core (13 nm) and satellite (3 nm) AuNPs with 80–90 DNA strands or satellite (5 nm) Au NPs with 2–3 DNA strands could be obtained, respectively. The impact of NP design and assembly on model cell system (J774A.1 murine macrophages) was further investigated. In the work, it was found that the CS Au NP (13 nm)‐AuNP (5 nm) superstructures showed sevenfold (5 nm) and twofold (3 nm) less uptake into macrophages compared with the 13 nm core AuNPs and the 5 nm satellite AuNPs, suggesting the design‐dependent uptake properties. Interestingly, the uptake of superstructures by macrophages monotonically decreased as a function of satellite to core ratio, demonstrating that the satellites blocked the macrophages from directly interacting with the core. The in vivo studies further demonstrated the current CS superstructures had much better tumor accumulation than the core NPs and non‐assembled mixture.

Based on the selective modification of AuNRs with DNA strands, Kotov and co‐workers designed the regiospecific plasmonic nanoassemblies using Au NRs and Au NPs (Figure 2c).^[^ ^93^ ^]^ In this work, the hexadecyltrimethylammonium bromide (CTAB) stabilized Au NRs were first synthesized. In order to selectively modify DNA onto the “End” or “Side” region of Au NRs, the CTAB concentration in the Au NRs solution was carefully controlled at ≈5 mm, at which concentration the CTAB density at the Au NR end region was much less than that at the side region. In this case, the Au NRs could be selectively modified with DNA strands only at the “End” region after addition of fixed thiol terminated DNA strands. For the “Side” modification, the “End” region was first modified with “Helper” DNA strand and the thiolated DNA strands could only be conjugated around the “Side” region. The selective modification was further confirmed by the regioselective AuNR‐AuNP CS nanoassemblies, where the complementary DNA‐AuNPs are only bound to the “End” or “Side” areas (Figure 2d). Interestingly, the assembled AuNR‐AuNP CS superstructures with various satellites demonstrated tailorable optical properties that could be used for biosensing.^[^ ^95^, ^96^ ^]^ A comprehensive strategy for regioselective functionalizing nanoparticles with DNA using a coating with diblock polymers was recently developed and applied for assembly a large family of cluster architectures.^[^ ^97^ ^]^ Gang group showed that 3D DNA architectures can be used stamping apparatus for the site‐specific molecular patterning of nanoparticles with single‐strand DNA^[^ ^98^ ^]^ and such NP can be assembled in precise clusters. Ability to create clusters is important for modulating optical properties. By creating chiral clusters form plasmonic particles their ability to rotate light can be also engineered.^[^ ^44^ ^]^ The specific cluster of plasmonic and light emitting nanoparticles, designed with DNA help, offer the ability to modulate both fluorescence and polarization of the emitted light,^[^ ^99^, ^100^ ^]^ which is extremely advantageous for biosensing and imaging applications.

In 2015, He et al. constructed Au‐UCNPs nanoclusters via DNA‐mediated assembly.^[^ ^101^ ^]^ In this work, Au NPs with plasmon resonances around 520 nm were selected to conjugate with silica (10 nm) coated UCNPs. The works showed that the upconversion luminescence (UCL) could be adjusted. After conjugation with Au NPs, an increase in both red and green emission peaks was observed at the ratio of 0.1:1 to 10:1 (AuNP:UCNP). However, a marked decrease in UCL was obtained at ratio higher than 10:1. Later, Wu et al. fabricated propeller‐like AuNR‐UCNP chiral tetramer assemblies by adopting a DNA‐driven strategy (Figure 2e).^[^ ^94^ ^]^ The chiral tetramers were constructed by the DNA‐Au NRs (aspect ratio of 3.3) and UCNPs (20 nm) with complementary DNA modification. For this, the side of Au NRs was conjugated with DNA1 at molar ratio of 1:1; the maleimide‐PEGylated UCNPs were modified with thiol DNA2 at the ratio of 1:3. To obtain tetramers, DNA1‐AuNRs were mixed with DNA2‐UCNPs at a ratio of 4:1. DNA hybridization resulted in the formation of propeller‐like tetramer assemblies, which was confirmed by the TEM images and 3D reconstruction TEM tomography (Figure 2f,g). In the study of chiral optical properties, a strong increase of circular dichroism (CD) bands within 600–800 nm from the tetramer assemblies was observed, which was attributed to the unique propeller‐like geometry of the NR‐UCNPs assemblies. The chiral optical properties UCL of the tetramers strongly depend on the distance (lengths of DNA sequence) between the AuNR and UCNPs, and Au NR aspect ratio. The UCL was strongly quenched when the length was shorter than 18 base pairs (bp), which could be ascribed to the nonradiative energy transfer to plasmonic state of Au NRs. The maximum UCL enhancement factor reached up to 21.3‐fold at the distance of 30 bp (≈10 nm). More importantly, the chiroptical and UCL properties of the AuNR‐UCNP tetramer assemblies enabled DNA detection with an unusual low limit of detection (13.2 am). In their following studies, they achieved ultrasensitive dual‐mode quantification of microRNA in living cells based on the DNA‐assembled Au‐UCNP nanoassemblies.^[^ ^102^, ^103^ ^]^

#### 3.2.2 Nanoparticle Superlattices

DNA nanotechnology provides a powerful tool for programmed self‐assembly of various building blocks into large superstructures, which has been widely explored recently. Through the programmable base‐pairing interactions, highly ordered crystalline assemblies are extensively explored in recent years by Mirkin, Gang, and others.^[^ ^9^, ^42^, ^104^, ^105^, ^106^, ^107^, ^108^, ^109^, ^110^ ^]^

**Figure 3:**
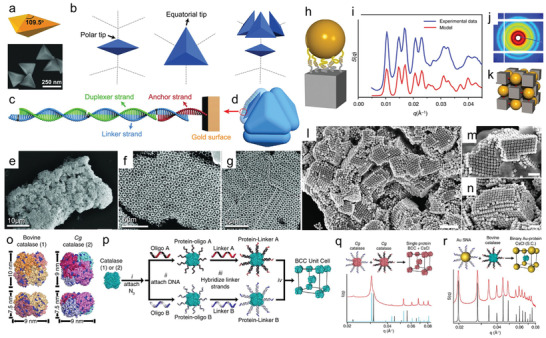
DNA‐mediated self‐assembly of clathrate colloidal crystals. a,b) The geometry and scanning electron microscopy image (SEM) of the Au trigonal bipyramids (TBPs). c,d) Illustration of the DNA linker design for the Au TBPs self‐assembly. e–g) The SEM and TEM images of the assembled TBP superlattices. Reproduced with permission.^[^ ^106^ ^]^ Copyright 2017, AAAS. Cube‐sphere hybrid nanoassembly superlattices. h,k) Scheme for the 46 nm cube and 46 nm sphere Au pair linked by DNA strands. i,j) SAXS results of the cube‐sphere superlattices. i–n) TEM images of the cube‐sphere superlattices. Reproduced with permission.^[^ ^105^ ^]^ Copyright 2015, Springer Nature. o,p) Synthesis and characterization of protein‐DNA conjugates. q) SAXS data for protein‐protein and r) protein‐AuNP superlattices. Reproduced with permission.^[^ ^84^ ^]^ Copyright 2015, National Academy of Sciences.

For the monary particle self‐assembly, Mirkin and co‐workers assembled triangular bipyramids (TBPs) into clathrate architectures (**Figure** **3**a–g).^[^ ^106^ ^]^ In their work, the uniform TBPs with ≈250 nm long edge and ≈177 nm short edge was first synthesized and then modified with 28‐base hexylthiol‐DNA strands. After purification, the modified TBPs were hybridized with various DNA strands (23 to 228 bases) which are all terminated with self‐complementary sticky end. The experimental studies showed that high‐quality TBP superlattices were formed by carefully controlling the hybridization temperature. This work demonstrates that the DNA template is a powerful tool for complex crystal self‐assembly. For the binary particle self‐assembly, Gang and co‐workers realized the large area self‐assembly of hetero‐shaped particles via the directional DNA bonds (Figure 3h–n).^[^ ^105^ ^]^ In their work, they developed 3D binary superlattices of cube‐sphere Au crystals. Both the small‐angle X‐ray scattering (SAXS) and scanning electron microscopy images confirmed the highly ordered structures of the assemblies. The interplay between particle shape and polymeric nature of DNA shell on a cubic particle can also result in unique organization of nanocubes with broken orientational symmetry in the unit cell.^[^ ^107^ ^]^

Proteins are the nature's most versatile and powerful nanoscale building blocks, and perform myriad functions in living systems.^[^ ^79^ ^]^ Therefore, a facile strategy for assembly of proteins into periodic structures, especially 3D crystalline protein structures, would have a wide range of applications. In 2015, Mirkin and co‐workers demonstrated 3D protein‐protein and AuNP‐protein binary crystalline nanostructures through the programmable ability of DNA‐DNA interactions (Figure 3o,p).^[^ ^84^ ^]^ In this work, the bovine and *Cg* catalases were first reacted with NHS‐N_3_ to functionalize the protein surface with azide groups, which were then conjugated to two distinct DBCO‐modified DNA single strands via copper‐free click reactions. Addition of linker DNA strands to DNA‐proteins followed by mixing two types of building blocks (DNA‐proteins, DNA‐AuNPs) would result in highly ordered assembly of the proteins into crystalline structures, which was also confirmed by the SAXS characterization (Figure 3q,r). Recently, Gang group demonstrated a general approach for organizing proteins into 3D lattices.^[^ ^111^ ^]^ In the developed method proteins functionalized with DNA, and integrated through a complementary hybridization with polyhedra DNA origami frame. These new building blocks, called material voxels, are then assembled into 3D lattices via hybridizations between vertices of DNA frames. The structure of formed ordered frameworks is fully determined by a frame shape, where tetrahedral, octahedra, and cubic frames were employed. Thus, the same or different kinds proteins and enzyme can be assembled in ordered 3D lattices.

#### 3.2.3 Dynamic Cluster Structures

**Figure 4:**
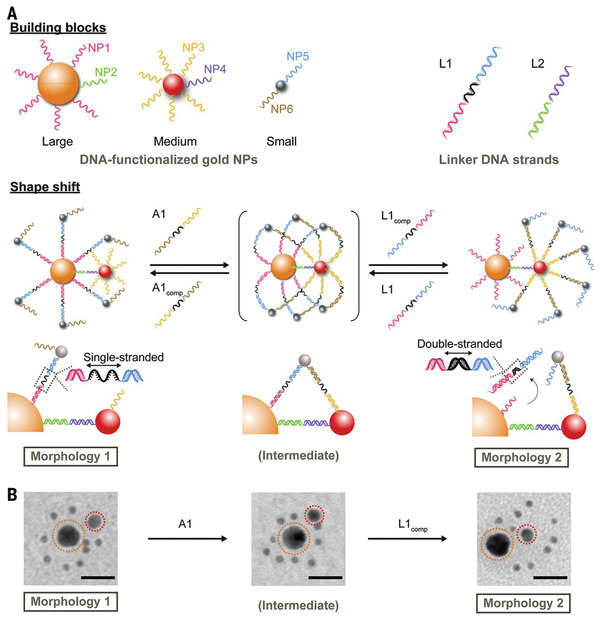
Scheme for the DNA‐controlled core‐satellite dynamic particle systems. a) The individual particle modification and its corresponding morphology changes in the presence of linker DNA. b) The corresponding TEM images of the particle assemblies mediated by DNA. Reproduced with permission.^[^ ^92^ ^]^ Copyright 2016, The American Association for the Advancement of Science.

According to base‐pairing interactions, DNA‐based particle superstructures with various degrees of organization and complexity of collective behavior are important for the development of responsive nanodevices or nanorobots. Recently, Chan and co‐workers reported a DNA‐controlled dynamic system for mediating the cellular interactions (**Figure** **4**).^[^ ^92^ ^]^ The system consisted of a core Au NP surrounded by small satellites, where the morphology transformation was realized in response to DNA via the toe‐hold displacement mechanism. In particular, the Au NP (13 nm) core was surrounded by one 6 nm Au NP and multiple 3 nm AuNPs, the conformation changed in response to DNA via the toe‐hold displacement reaction, which then altered the optical properties and biological interactions of the assembles. In morphology 1, the targeting ligand folic acid (FA) was hindered by 3 and 6 nm satellites, resulting in little targeting ability (OFF state). In the presence of linker DNA, the nanoassemblies could transfer into morphology 2, and the “hidden” FA was exposed to the microenvironment through the relocation of 3 nm AuNPs, activating the targeting ability of FA (ON state). The in vitro experiments further revealed that the cellular uptake of FA functionalized nanoassemblies with morphology 2 was 2.5 times higher than that of morphology 1.

### 3.3 DNA Origami‐Templated Particle Assembly

Structural DNA nanotechnology provides a powerful tool for tailoring various nanoscale hierarchical architectures via the Watson–Crick base pairing interaction. Among these, well‐designed DNA origami and DNA scaffold structures ranging from 10–100 nm have been developed very recently.^[^ ^11^, ^112^, ^113^, ^114^, ^115^, ^116^, ^117^, ^118^, ^119^, ^120^ ^]^ Typically, the DNA origami and scaffold structures are assembled by using M13 bacteriophage genome DNA strand and staple strands or multiple short DNA strands. Due to their intrinsic features, such DNA nanostructures could be easily programmed for precise anchoring of various functional NPs,^[^ ^44^, ^121^, ^122^, ^123^, ^124^, ^125^ ^]^ protein,^[^ ^6^, ^126^, ^127^, ^128^ ^]^ or small molecules.^[^ ^122^, ^129^, ^130^ ^]^ Due to the strong base‐pairing interaction, the DNA could be utilized for constructing 1D, 2D, and 3D nanostructures with precision. For example, 1D DNA tiles (or scaffold) could be easily prepared by mixing two complementary single DNA strands.^[^ ^126^ ^]^ By using a “bottom‐up” self‐assembly method, Rothemund first fabricated arbitrary 2D shapes of DNA origami nanostructures.^[^ ^11^ ^]^ In his pioneering work, he developed a versatile and simple one‐pot method to prepare various DNA origami nanostructures by mixing a number of short single DNA strands with a M13mp18 genomic DNA strand. Later, more complex shapes (such as 3D DNA origami, twist or curve DNA nanostructures) were reported.^[^ ^16^, ^131^ ^]^

**Figure 5:**
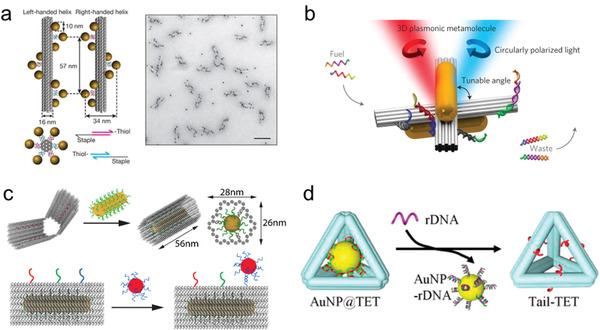
a) Scheme of left‐ and right‐handed nanohelices assembly by DNA helix bundles and nine AuNPs (left) and the corresponding TEM image of left‐handed Au nanohelices (right). Reproduced with permission.^[^ ^134^ ^]^ Copyright 2012, Springer Nature. b) Schematic diagram of 3D plasmonic metamolecule assembled by two switchable DNA origami templates and two AuNRs. Reproduced with permission.^[^ ^136^ ^]^ Copyright 2014, Springer Nature. c) Site‐specific binding of AuNRs and AuNPs onto DNA origami clamps. Reproduced with permission.^[^ ^121^ ^]^ Copyright 2015, American Chemical Society. d) Release of AuNPs from the DNA tetrahedral nanocages. Reproduced with permission.^[^ ^142^ ^]^ Copyright 2014, American Chemical Society.

Recently, the emerging DNA origami technology provides new approaches for direct self‐assembly of NPs and other species into well‐ordered DNA nanostructures with complex geometries. In 2008, Yan and co‐workers reported the first example of self‐assembly of semiconducting NPs (QDs) into 2D periodic nanoarrays by using DNA tile template.^[^ ^132^ ^]^ In their studies, the core‐shell QDs (CdSe/ZnS) were modified with streptavidin, and the DNA tile was modified with biotin. According to the specific binding interactions between the streptavidin and biotin, the QDs would be organized onto the DNA tile arrays accurately. Subsequently, various 3D DNA tubules were fabricated by the same group through self‐assembly of sDNA‐AuNPs and 2D DNA tile.^[^ ^133^ ^]^ Using the DNA origami template, AuNPs helices and various planet‐satellite nanoclusters with unique optical properties were also successfully fabricated by Liedl and co‐workers (**Figure** **5**a).^[^ ^122^, ^134^, ^135^ ^]^

In 2014, Kuzyk et al. created reconfigurable 3D plasmonic metamolecules using DNA‐regulated conformational changes at nanoscale levels (Figure 5b).^[^ ^136^ ^]^ In the 3D metamolecules, two AuNRs were held on a switchable DNA origami which was constructed by two connected bundles. Interestingly, the relative angel between the two AuNRs could be altered with the aid of the specifically designed DNA strands, which could work as the fuel to drive the relative angle changes through toe hold‐mediated strand displacement reaction. In addition, left‐ and right‐handed plasmonic metamolecules could be easily realized by adding the corresponding DNA strand, which was further confirmed by the TEM characterization and CD spectra measurement. Later, the pH‐responsive plasmonic metamolecules were realized by the same group by utilizing DNA tile containing CGC and TAT triplets through Watson–Crick and Hoogsteen interactions.^[^ ^137^ ^]^ Gang group's studies showed that Hoogsteen interactions can be controlled by pH at the lipid interfaces, and that permits switching 2D lattice arrangements.^[^ ^138^ ^]^ The pH‐responsive structures would have great potential in controlled drug delivery.

Hung et al. constructed spatially ordered 2D nanoarrays by directional self‐assembly of 5 nm DNA‐AuNPs on triangular DNA origami through corner‐selective binding.^[^ ^139^ ^]^ Similarly, DNA functional Ag NPs could be selectively conjugated at the specific positions on the DNA origami with high fidelity.^[^ ^140^ ^]^ In 2016, Shen et al. reported AuNRs functionalized DNA clamp nanostructures using a rectangular DNA origami template (Figure 5c).^[^ ^121^ ^]^ The set of capture strands on the outside of the DNA clamp was used for constructing a series of designed hybrid nanostructures, which created specific binding sites for attachment of Au NPs on the top, middle, and bottom of the clamp. Using the DNA origami template, AuNR@AuNP helices nanostructures with tunable chiroptical response were prepared, which provided a facile method for bottom‐up construction of hybrid nanoarchitectures.^[^ ^141^ ^]^

Rational assembly of optical NPs into 2D or 3D functional devices, which possess the unique features of DNA nanostructures and physical/chemical properties of the NPs, are of great importance in the field of photonics, metamaterials, and biotechnology. Recently, Mao and co‐workers developed a class of core‐shell AuNP@DNA complex, where the 5 nm Au NPs were encapsulated in the well‐defined DNA nanocages (length: ≈14 nm) (such as DNA tetrahedron, octahedron, and icosahedron) (Figure 5d).^[^ ^142^ ^]^ In their work, the Au NPs were swallowed into the central cavities of preassembled DNA wireframe nanocages through hybridization between the DNA single strand tails on the nanocages and AuNPs. The encapsulated Au NPs could be released upon the addition of excessive amount of complementary DNA single strands. Alternatively, methane (CH_4_) molecule‐like tetrahedral nanostructures could also be constructed by modification of the caged AuNPs with two different DNA single strands.^[^ ^40^ ^]^ Briefly, the first type of DNA single strands on the surface of inside Au NPs were complementary to the DNA strand tails on the nanocages, while the second one is complementary to that located on the outside AuNPs. Similarly, other types of polyhedral nanostructures could also be realized, including octahedral SF_6_‐like, trigonal prismatic W(CH_3_)_6_‐like, dual‐core ethane (C_2_H_6_)‐like nanostructures.

**Figure 6:**
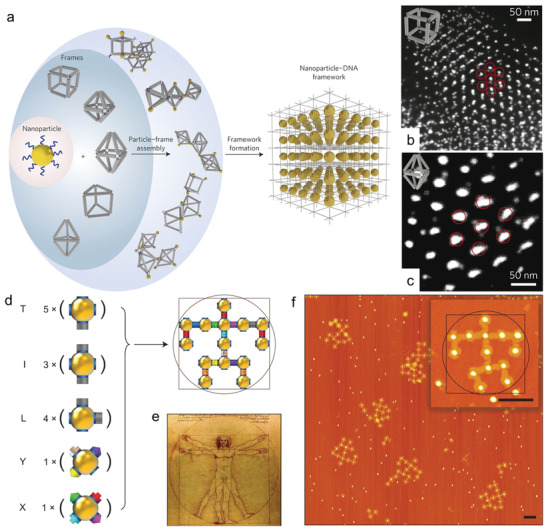
a) Illustration for the lattice assembly via the NP‐DNA frameworks. b,c) Cryo‐STEM images of cubic and body‐centered‐tetragonal (BCT) superlattices. Reproduced with permission.^[^ ^143^ ^]^ Copyright 2016, Springer Nature. d) Scheme of the architecture of nanoscale Leonardo da Vinci's "Vitruvian Man". e) Leonardo da Vinci's "Vitruvian Man". f) Typical AFM image of the assembled nanoscale Leonardo da Vinci's "Vitruvian Man". Reproduced with permission.^[^ ^14^ ^]^ Copyright 2016, Springer Nature.

Excitingly, the similar strategy can be used for construction of ordered 3D superstructures. In this field, Gang groups proposed and implemented the self‐assembly of particle superlattices through NP‐DNA wireframe.^[^ ^14^, ^40^, ^44^, ^143^, ^144^ ^]^ Different types of 3D superlattices were demonstrated in their recent works, which employed the rigid tetrahedral DNA origami nanocages (with or without AuNPs encapsulation) as the building blocks (**Figure** **6**a–c).^[^ ^144^ ^]^ The vertex of each tetrahedral nanocage was installed with sticky patch DNA strands for tetravalent binding to the basis NPs. Diamond superlattices were finally realized, and unique superlattice structures were also confirmed by cryogenic EM and SAXS measurement. Besides tetrahedral DNA origami nanocages, other kinds of DNA origami polyhedral cages (such as cube, prism, octahedron, TBP, elongated square bipyramid) were also suitable as the building blocks to construct linear 2D nanoarrays and 3D crystallographic superlattices (Figure 6d,e).^[^ ^44^, ^143^ ^]^ In addition, the DNA‐directed self‐assembly strategy demonstrated its versatility for the fabrication of arbitrary targeted architectures. For example, diverse planner architectures like nanoscale model of Leonardo da Vinci's "Vitruvian Man" can be fabricated with the integration of NPs and DNA origami frames (Figure 6f).^[^ ^14^ ^]^ The above studies greatly broadened the utilization of DNA as a powerful tool for precise tailoring and organizing complex architectures with high fidelity.

In addition to inorganic NPs, the biomolecules (such as proteins and enzymes) can also be well‐tailored on the DNA origami at nanoscale levels. For this, Yan and co‐workers first developed a multi‐helix DNA tile for precise and selective binding of two different proteins.^[^ ^6^ ^]^ With the aid of distance precision controlling, the effect of enzyme distance (such as GOx and HRP) on the cascade reactions was evaluated in the DNA tile based hybrid nanosystems,^[^ ^126^, ^145^ ^]^ and assembled in 3D‐organized GOx‐HRP cascades.^[^ ^111^ ^]^ The recent detailed studies revealed that DNA origami can affect local pH environments of enzyme which affect its activity.^[^ ^146^ ^]^

## 4 Biomedical Applications of Self‐Assembled DNA Nanostructures

DNA nanotechnology provides a strategy for programmed self‐assembly of arbitrary hierarchical nanoassemblies with nanoscale precision, which provides attractive opportunities for biosensing, imaging, drug delivery, and cancer therapy.^[^ ^17^, ^18^, ^147^, ^148^ ^]^ Recently, external stimuli‐responsive NP platforms for biomedical applications have received increasing attention, which could effectively increase cargo accumulation at the targeted areas, largely decrease the side‐effect, and avoid under‐ or over‐dosing.^[^ ^149^, ^150^, ^151^, ^152^, ^153^ ^]^ In this part, various biomedical applications of the assembled DNA nanostructures will be discussed in detail.

### 4.1 DNA Nanostructures for Biosensing

Nucleic acid detection (DNA, RNA) is very important for both fundamental and clinical studies in the biomedical fields. Recently, various strategies have been proposed for the rapid detection of nucleic acids with high accuracy, among which, the DNA nanotechnology is one of the promising methods. For example, Su et al. prepared thiolated DNA‐based CdTe/CdS core‐shell QDs system for DNA and miRNA detection.^[^ ^154^ ^]^ In their work, 3‐mercaptopro pionic acid (MPA) stabilized CdTe/CdS core‐shell QDs were first prepared and subsequently conjugated with thiolated DNA strands via ligand exchange. The conjugation of DNA was confirmed by UV–vis and agarose gel electrophoresis. In the presence of target DNA (partially complementary to thiol‐DNA and BHQ_2_‐DNA) and an organic quencher (BHQ_2_)‐labeled DNA strands, the fluorescence of QDs would be decreased with the increase of the target DNA concentrations based on Forster resonance energy transfer (FRET). Inspiringly, the detection limit of the target could reach as low as 1 and 10 fm for DNA and miRNA, respectively.

He et al. reported DNA‐programmed dynamic structures of multicolor QDs as another kind of FRET‐based system.^[^ ^155^ ^]^ In their design, three monovalent DNA‐QDs (A:ZnHgSe, B:CdTe, and C:ZnCdSe) were assembled into ternary QDs complex with the aid of DNA template. The three QDs had well separated fluorescence emission (red for ZnHgSe, green for CdTe, blue for ZnCdSe). Based on the spectral overlap, FRET occurred in the ternary QDs complex with high efficiency of 81.6% (blue to green), 76.5% (green to red), and 82.3% (blue to red). In the presence of single strand fuel DNA (containing two different toeholds at both ends), the QDs A could drop off. The disassembly of QD A resulted in notable green fluorescence increase of QD B and red signal decrease of QD A, which showed the RFET between QD A and B was off. In addition, the FRET between A and B could be restored when anti‐fuel DNA strands were added to reassemble the ternary QDs complex. Similarly, the disassembly and reassembly of QD C in the ternary QDs complex could also be adjusted accordingly to change the FRET between QD B and QD C. Such strategy is suitable for logical operations and has great promise for intelligent sensing.

Kuang and co‐worker demonstrated that the DNA directed CS Au NR‐UCNP nanoassemblies could simultaneously detect two different miRNA cancer markers (miR‐21 and miR‐200b).^[^ ^103^ ^]^ In their work, six DNA strands were used to construct the CS structures. Especially, the end and side of the AuNRs were conjugated with two different thiol‐DNA strands, which could partially hybridize with another two DNA‐modified UCNPs at the end and side, respectively. For dual miRNA detection, TAMRA and Cy5.5 (dye molecules) were conjugated with fifth and sixth ssDNA, which would partially hybridize on the end and side of the AuNRs. In this system, the fluorescence of the TAMRA (excitation: 559 nm) and Cy5.5 (excitation: 750 nm) were quenched by the AuNRs. While, in the presence of miR‐21, it would recognize sequence on the DNA linked to the end of AuNRs, leading to the release of UCNPs from the end. Therefore, the upconversion fluorescence was recovered and TAMRA would be lighted by the green emission of the UCNPs. In the presence of miR‐200b, the Cy‐5.5 would be excited by the red emission of the released UCNPs. When both miR‐21 and miR‐200b were present, the display emission of TAMRA and Cy5.5 were all recovered. Using this technique, the limit of detection of 3.2 zmol ng_RNA_^−1^ and 10.3 zmol/ng_RNA_ for miR‐21 and miR‐200b were realized. More importantly, zeptomolar sensitivity was also achieved both in living cells and in vivo assays, demonstrating its promising in biological and medical analysis. Similarly, Zhao et al. demonstrated that an activatable UCNP‐based DNA nanodevice for the detection of miRNAs in vitro and in vivo.^[^ ^150^ ^]^

**Figure 7:**
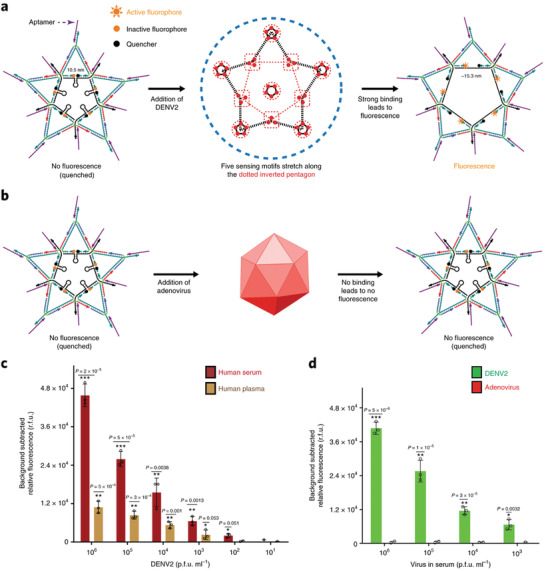
a,b) Scheme of the DNA star‐aptamer sensor system. c) The detection of DENV in plasma and human serum. d) The star sensor could not detect the adenovirus in serum. Reproduced with permission.^[^ ^156^ ^]^ Copyright 2020, Springer Nature.

Kwon et al. recently developed a star‐shaped DNA scaffold nanostructure, which carried five molecular beacon‐like motifs, for viral sensing and inhibition with precise and multivalent spatial pattern‐recognition (**Figure** **7**).^[^ ^156^ ^]^ In their work, the star‐shaped DNA scaffold acted as the template to display ten dengue envelope protein domain III (ED3)‐targeting aptamers, which well matched the spatial arrangement of the ED3 clusters on the dengue (DENV) viral surface. In the star‐aptamer complex system, the aptamer‐DENV binding strength increased when the aptamers were placed on the DNA scaffolds along with the increasing match of the ED3 sites on DENV with the DNA star. To form a viral sensor, the 6‐FAM (fluorophore) and BHQ‐1 (quencher) terminated DNA strands were hybridized to the inner edge. In the presence of DENV, the FAM fluorophores and the BHQ‐1 quenchers would separate to turn on the fluorescence, which gave a readout of the target nucleic acid hybridization events. Their studies demonstrated that the DNA star‐aptamer sensor could directly detect the DENV at a very low detection limit of 1 × 10^3^ and 1 × 10^2^ p.f.u. mL^−1^ in plasma and human serum, respectively. Considering the steric hindrance effect of DNA nanostructures, Tan and co‐workers reported a DNA molecular sieve for size‐selective discrimination of mature microRNA and precursor microRNA in living cells.^[^ ^157^ ^]^

Due to the strong noncovalent binding between the ssDNA and 2D nanomaterials, the 2D nanomaterials (such as graphene, transition metal dichalcogenides, g‐C_3_N_4_, MOFs, and covalent organic frameworks) have been widely utilized for DNA detection recently.^[^ ^158^ ^]^ For example, Yang and co‐workers first reported the graphene oxide (GO)‐based nanoprobe for the detection of biomolecules.^[^ ^159^ ^]^ In their study, dye labeled the single strand DNA (ssDNA) was mixed with the GO, where the fluorescence of the dye was quenched by the GO. At the presence of targeting DNA, the ssDNA would hybridize with it and release the ssDNA, leading to the restoration of the fluorescence. The change of the fluorescence could be used for quantitative detection of DNA. Inspired by the above works, kinds of other 2D nanoprobes have been constructed for biosensing.^[^ ^160^, ^161^, ^162^ ^]^

### 4.2 DNA Nanostructures for Bioimaging

Precise recognition of the diseased tissues is the prerequisite for diagnosis and therapy. Numerous noninvasive and invasive diagnostic methods have been developed for such purposes. Of these, optical imaging is a promising imaging modality due to the low cost and convenience.^[^ ^163^, ^164^ ^]^ In the past decades, DNA based nanostructures with high level of structural programmability have been developed in such particular application, due to their high purity, reproducibility, biocompatibility, and biodegradability.^[^ ^165^ ^]^

**Figure 8:**
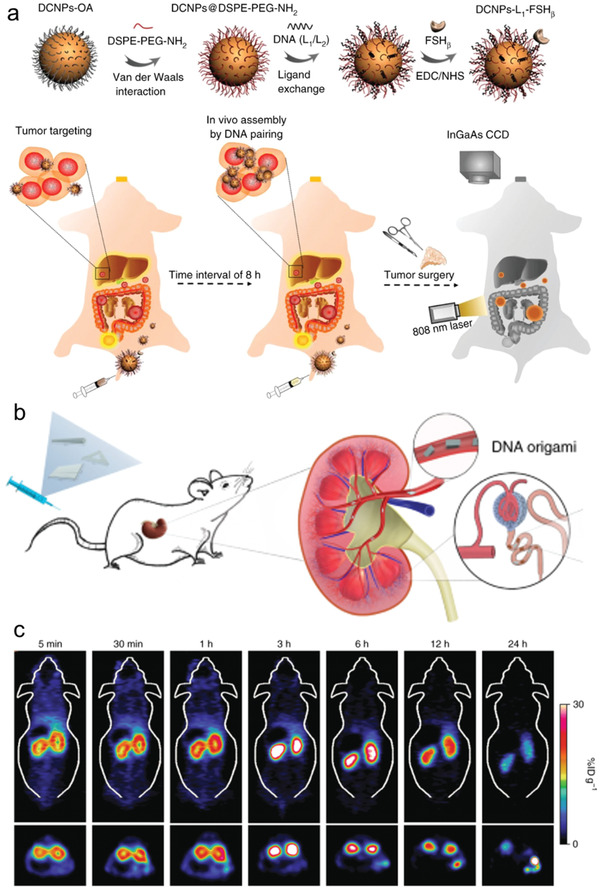
a) Scheme of the DCNPs‐based NIR‐II nanoprobes. Reproduced with permission.^[^ ^166^ ^]^ Copyright 2018, Springer Nature. b) Scheme of the preferential renal accumulation of the DNA origami nanostructures enabled protection against acute kidney injury (AKI). c) Positron‐emission tomography (PET) images of ^64^Cu‐labeled DNA origami nanostructures in the kidneys of mice with AKI. Reproduced with permission.^[^ ^170^ ^]^ Copyright 2018, Springer Nature.

Wang et al. reported rare‐earth doped NP‐based NIR‐II downconversion (DCNPs) nanoprobes for image‐guided surgery (**Figure** **8**a).^[^ ^166^ ^]^ In this work, the batches of DCNPs were modified with DNA strands and the complementary DNA strands. After two‐staged sequence injection of the nanoprobes, the two complementary DNA single strands modified DCNPs would assemble into larger clusters in vivo. Interestingly, the experimental results showed that in vivo self‐assembly of the DCNPs nanoprobes would increase their accumulation and retention in the tumor areas. The maximum NIR‐II fluorescence in the tumor area could be clearly observed at 12 h post‐injection, while the retention of nanoprobes in liver decreased dramatically, indicating its rapid hepatic and renal clearance. Such a strategy provides optimal NIR‐II imaging with a deeper tissue penetration. Later on, Lu et al. reported a pH‐responsive i‐motif DNA assembled iron oxide (≈3 nm) nanocluster assemblies for highly sensitive diagnosis of small hepatocellular carcinoma.^[^ ^167^ ^]^ At the acidic tumor microenvironment, the iron oxide nanocluster assemblies would disassemble into discrete iron oxide particles, thus converting the T2 to T1 contrast agent. The design provided a strategy to distinguish the normal liver and early‐stage small hepatocellular carcinomas.

Tian and co‐workers reported a triangular DNA origami‐AuNR hybrid nanostructure platform for efficient optoacoustic imaging (OAI) and cancer therapy.^[^ ^168^ ^]^ To fabricate the hybrid DNA nanostructures, capture strands were designed on triangular DNA origami to provide binding sites for complementary DNA modified AuNRs (42 × 12 nm). The in vitro experiments showed that the DNA origami‐AuNR hybrids had a higher accumulation than that of AuNRs alone. At 3 h post‐injection, the OAI signal could be observed from the AuNRs and DNA origami‐AuNR hybrids treated tumor‐bearing mice, respectively. Interestingly, the signal corresponding to DNA origami‐AuNR hybrids gradually increased starting from 3 h and remained high for at least 24 h, which was very different from AuNRs treated mice. In addition, the presence of the AuNRs in DNA origami‐AuNR hybrids made it a photothermal agent for tumor inhibition. The above discoveries demonstrated the DNA origami nanostructures could efficiently optimize the internalization of imaging probes in cancer diagnostics.

Recent studies showed that the DNA origami nanostructures alone have great promise for bioimaging.^[^ ^169^, ^170^, ^171^, ^172^ ^]^ In particular, Anderson and co‐workers constructed oligonucleotide NPs (OPNs) for targeted imaging and small interfering RNA delivery in mice.^[^ ^169^ ^]^ In this work, the OPNs had a monodispersed size of ≈10 nm (30 bp per edge) and was assembled by six complementary oligonucleotides. In the OPNs system, there were six overhangs on each edge, of which three of them were used for conjugating with FA and the other three for anchoring fluorescence dye (Cy5). In comparison with various peptides and FA, the FA‐OPNs exhibited the highest gene silencing ability. Interestingly, the controllable spatial orientation of siRNA and density of the targeting ligands on the OPNs facilitated enhanced cell internalization and gene silencing. Moreover, the in vivo efficiency of the nanoplatform was also confirmed by administration of the Cy5‐labeled FA‐ONPs into KB tumor bearing mice. The fluorescence molecular tomography fused with computed tomography results showed the OPNs mainly accumulated in the tumor and kidneys, with no detectable signals in other organs. Further pharmacokinetic study demonstrated that the OPNs siRNA delivery vehicle had a longer blood circulation time (*t*_1/2_ = 24.2 min) than siRNA alone (*t*_1/2_ = 6 min). Later on, Ahn and co‐workers developed fluorescence‐labeled DNA tetrahedron NPs (DTNs) for sentinel lymph nodes (SLNs) imaging.^[^ ^173^ ^]^ Their in vivo studies revealed that the DTNs demonstrated longer retention time and enhanced translocation in SLNs in the node. Ding and co‐workers directly visualized the tubular DNA origami intracellular distribution in MCF‐7 cells by using a label‐free fluorescence probe.^[^ ^174^ ^]^ In their work, the tubular DNA origami nanostructures provided the docking sites for carbazole‐based cyanine fluorophores to form a DNA‐probes complex with strong fluorescence induced by restriction of intramolecular rotation. Before binding with DNA origami, the probes had weak fluorescence.^[^ ^175^ ^]^ When the complex was administered into MCF‐7 cells, the fluorescence of the DNA‐probes was turn‐on which could be used for intracellular tracking of the NPs. The in vitro results showed that the DNA origami was mainly located in lysosome at 12 h and was dissociated after 60 h incubation.

Fan and co‐workers constructed multiarmed DNA tetrahedral nanostructures (DTN) for dual‐mode in vivo imaging.^[^ ^164^ ^]^ The functional components such as tumor‐targeting FA, NIR emitter (Dylight 755), and radioactive isotope were precisely anchored on the DTN at the prescribed positions. Compared with double strand (dsDNA), the DTN demonstrated longer circulation time in the mice. The functional components enabled the DTN as a NIR fluorescence/single‐photon emission computed tomography dual‐mode imaging agent. Recently, Cai and co‐workers reported that the radiolabeled DNA origami nanostructures demonstrated preferential accumulation in both kidneys of healthy mice and mice with acute kidney injury (AKI) (Figure 8b).^[^ ^170^ ^]^ In this work, the positron‐emission tomography (PET) scanning was used to monitor the biodistribution of ^64^Cu‐labeled DNA origami nanostructures. The experimental results showed that the ^64^Cu‐labeled rectangular DNA origami nanostructures rapidly accumulated in the kidneys at 5 min post‐injection in the AKI mice. More interestingly, it gradually accumulated in the bladders, suggesting that the injured kidneys still excreted the DNA origami nanostructures.

### 4.3 DNA Nanostructures for Drug Delivery

Due to the high structural programmability, high biocompatibility, tailorable shape, and size, the emerging DNA nanostructure carriers have been widely explored for drug delivery and disease therapy.^[^ ^18^, ^151^, ^163^, ^176^, ^177^ ^]^ As a template, the DNA origami alone could be used for anticancer drug delivery. For example, Ding and co‐workers reported the doxorubicin (DOX) could be non‐covalently attached to the triangle DNA origami nanostructures through intercalation.^[^ ^178^ ^]^ The loading efficiency in the DNA origami was reached up to 50–60%. The DOX‐origami complex demonstrated prominent cytotoxicity both to regular human breast adenocarcinoma cancer cells (MCF7) and DOX‐resistant cancer cells. This work suggested that the DNA origamis have great potential to act as a kind of biocompatible delivery vehicle.

**Figure 9:**
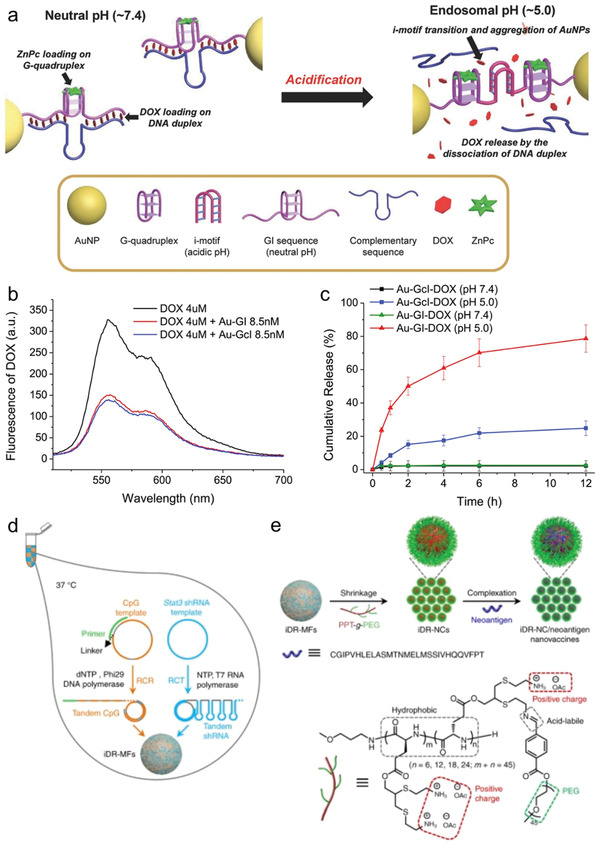
a) Illustration of the DNA‐AuNP dynamic nanomachine for cargo delivery. b,c) Release profile of the DOX from DNA‐AuNP dynamic nanomachine. Reproduced with permission.^[^ ^179^ ^]^ Copyright 2018, Wiley‐VCH GmbH. d,e) Scheme for the synthesis of iDR‐NC nanovaccines. Reproduced with permission.^[^ ^183^ ^]^ Copyright 2017, Springer Nature.

In a recent study, Park et al. developed an anticancer drug and photosensitizer coloaded DNA‐AuNP dynamic nanomachine for cargo delivery and synergistic cancer therapy (**Figure** **9**a).^[^ ^179^ ^]^ In this work, the AuNPs were conjugated with pH‐responsive i‐motif and G‐quadruplex DNA strands, where the DOX was intercalated into duplex DNA and the ZnPc was selectively loaded on the G‐quadruplex via *π*–*π* interaction. In the acidic condition, the formation of the i‐motif DNA structures would lead to the aggregation of the AuNPs and DOX release by the dissociation of the DNA duplex, demonstrating a pH‐responsive drug release behavior (Figure 9b–f). Upon light irradiation, notable heat and a large amount of ^1^O_2_ could be generated. Moreover, the synergistic effects of chemo, photodynamic, and photothermal effect were demonstrated in the in vitro and in vivo studies. Similarly, Raeesi et al. developed a DNA‐AuNR superstructure platform for controlled drug loading and release by tuning the length of the drug loading zone.^[^ ^90^ ^]^

Very recently, Jiang and co‐workers reported a polymeric spherical nucleic acid (SNA) system programmed with high loading capacity of therapeutic agents via super sandwich hybridization reaction for bioimaging.^[^ ^180^ ^]^ In the polymeric SNAs system, a long concatemer DNA strand was conjugated onto the AuNPs, which was self‐assembled by two different short alternating DNA strands (P1 and P2) and the immobilized capture probes (CP) on the AuNPs. The P1 was labeled with Ce6‐aptamer sequence which could bind to ATP specifically, and the P2 was labeled with a BHQ_2_ quencher where the strand was partially complementary to P1. In the system, the long concatemer dsDNA was constructed by the periodically repeated units of P1 and P2 based on partial hybridization. The GC pairs were used for DOX loading, which would result in the quenching of the fluorescence. In the absence of ATP, the singlet oxygen (^1^O_2_) generation from Ce6 was quenched by BHQ_2_. While the fluorescence of both Ce6 and DOX could be recovered once the polymeric SNAs were endocytosed by the HeLa cells (ATP concentration around 1–10 mm), resulting from the stronger binding of ATP with Ce6‐P1‐aptamer. This ATP‐responsive system demonstrated a promising imaging‐guided drug release platform for activatable cancer therapy.

Such a drug delivery strategy is not only suitable for other kinds of DNA‐NPs hybrid nanostructures, but also for the DNA origami nanostructures. For this, Huang and co‐workers developed a DNA icosahedra nanostructure as the anticancer drug carrier for cancer therapy.^[^ ^181^ ^]^ In their study, the DNA icosahedra nanostructures were assembled by sticky ended five or six‐point‐star motif via the sticky‐end association between the tiles. The anticancer drug DOX was incorporated into the DNA icosahedra nanostructures, where the dsDNA containing ‐CG‐ base pairs served as DOX docking sites. The in vitro experimental results revealed that the DOX‐loaded MUC‐1 aptamer‐DNA icosahedra hybrid nanostructures demonstrated enhanced cellular internalization in the MUC‐1 positive MCF‐7 cells, compared with free DOX or untargeted DOX loaded DNA icosahedra nanostructures. This work showed that the DOX‐intercalated DNA origami nanostructures had great promise in drug delivery. Later, Högbery and co‐workers introduced the tubular‐shaped DNA origami nanostructures based drug delivery systems for three different cancer cell lines (MDA‐MB‐231, MDA‐MB‐468, MCF‐7) therapy.^[^ ^182^ ^]^ They designed two different DNA origami nanostructures with varying degrees of global twist for tuning the drug encapsulation efficiency and release rate. Interestingly, the drug encapsulation efficiency and release rate could be tuned by tailoring the DNA origami design. Compared with the DOX alone, the DOX‐loaded tubular DNA origami demonstrated increased cytotoxicity and lower intracellular elimination rate. In particular, the drug release kinetics could be controlled and tuned through changing the degrees of twist.

Recently, DNA nanostructures were investigated as the vehicles for efficient delivery of CpG in vivo.^[^ ^183^, ^184^, ^185^, ^186^, ^187^, ^188^ ^]^ However, the low efficacy and instability of CpG ODNs limit their applications. Therefore, various carriers have been explored for efficient delivery of CpG. Among them, the DNA‐based and DNA origami nanostructure‐based delivery systems were developed. For example, Fan and colleagues developed an organelle‐responsive MOF‐based release system for specific delivery of immunostimulatory DNA.^[^ ^189^ ^]^ In the system, the cytosine‐phosphate‐guanosine (CpG) strands were adsorbed onto UiO‐66‐NH_2_ NPs through coordination binding between the phosphate and the unsaturated zirconium centers, and MOF carriers were further protected by calcium phosphate (CaP) exoskeleton. After endocytosis by the cells, the CaP shell would dissolve in the acidic microenvironment and efficiently generate phosphate ions, resulting in the release of CpG.

Fan and co‐workers introduced a tetrahedral DNA origami nanostructure with unmethylated CpG encapsulation for efficient delivery of CpG.^[^ ^184^ ^]^ In their work, the CpG loaded tetrahedral DNA nanostructures demonstrated enhanced stability and could be efficient internalized by macrophage‐like RAW264.7 cells with no transfection agents. After uptake by cells, the CpG ODNs were recognized by TLR9 to secret high‐level of pro‐inflammatory cytokines (including tumor necrosis factor, interleukin‐6, and interleckin‐12), showing enhanced immunostimulatory effect of the DNA nanostructures. Based on similar strategy, Liedl and co‐workers developed another DNA origami carrier for CpG ODNs loading and immunostimulation.^[^ ^190^ ^]^ They utilized a 30‐helix DNA origami tube that acted as a vehicle for CpG loading. Each origami tube contained 62 CpG ODNs at the predesigned binding sites. The in vitro experiments showed the CpG ODNs conjugated DNA origami tubes displayed improved loading efficiency and cellular internalization, demonstrating enhanced immunostimulatory activities. Due to the programmability and biocompatibility of structural DNA nanostructures, other shaped nanostructures have also been developed for immunotherapy.^[^ ^191^ ^]^

Chen and co‐workers recently developed self‐assembled intertwining DNA‐RNA nanocapsules that could efficiently deliver the CpG, short hairpin RNA (shRNA), and tumor‐specific peptide neoantigens to the antigen presenting cells (APCs) for cancer immunotherapy (Figure 9d,e).^[^ ^183^ ^]^ In this work, the nanovaccines were produced by tandem CpG and shRNA via concurrent rolling circle replication and rolling circle transcription, which was later self‐assembled into DNA‐RNA microflowers. By using PEG (polyethylene glycol)‐grafted cationic polypeptides, the microflowers would shrink into DNA‐RNA nanocapsules for neoantigen loading. The experimental studies showed the DNA‐RNA nanocapsules were efficiently delivered into APCs in the lymph nodes, eliciting higher level of neoantigen‐specific T cell response and synergistic tumor inhibition ability.

### 4.4 DNA Nanostructures for Disease Therapy

The high level of structural versatility and biocompatibility enable the DNA nanostructures as a synthetic host for the delivery of large NPs,^[^ ^192^, ^193^, ^194^ ^]^ and proteins^[^ ^195^, ^196^, ^197^, ^198^, ^199^, ^200^ ^]^ for effective cancer therapy. Inspired by DNA hybridization, Farokhzad and co‐workers reported a DNA‐based AuNRs platform that could be used as a NIR‐responsive targeted agent for cancer therapy.^[^ ^201^ ^]^ This platform is composed of three functional components: AuNRs (50 × 10 nm), CG bp rich‐thiolated DNA strands, and a PEG layer. The CG bp rich‐DNA strands provided the loading sites for DOX. In order to serve as NIR‐responsive drug‐loading carriers, another complementary DNA strand with cell‐specific targeting ligand modification was used. Upon NIR light irradiation, the AuNRs would produce heat and de‐hybridize the DNA double helix, leading to the triggered release of DOX at the targeted sites for chemotherapy. The presence of PEG layers would prolong the NP circulation time in vivo. Both the in vitro and in vivo experiments demonstrated the DNA‐based platform selectively delivered the anticancer drugs to the targeted KB and HeLa cells, resulting in efficient tumor growth inhibition through synergistic photothermal/chemotherapy. In particular, the drug loading (DOX) efficiency could be easily tuned by changing the length of ‐CG‐ base pair loading zone.^[^ ^90^ ^]^

**Figure 10:**
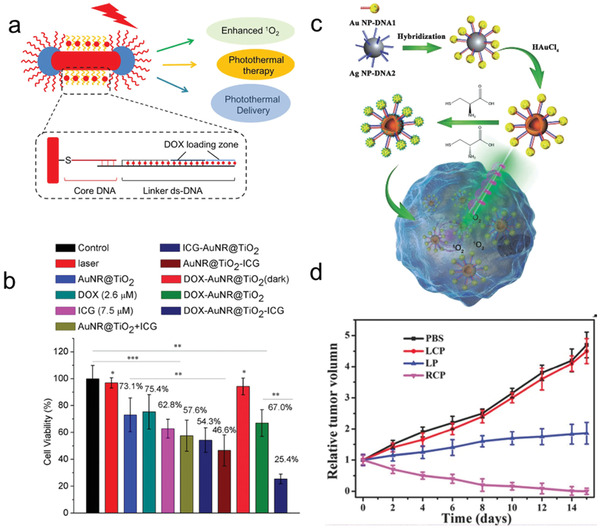
a) Scheme of TiO_2_‐capped AuNRs for plasmon‐enhanced of ^1^O_2_ generation and photothermal delivery of therapeutic agents. b) Quantitative analysis of MDA‐MB‐468 cell viabilities with different treatments. Reproduced with permission.^[^ ^202^ ^]^ Copyright 2018, American Chemical Society. c) Scheme of the self‐assembled shell‐satellite Ag‐Au nanostructure acted as a chiral photodynamic agent. d) The relative tumor growth curves after various treatments. Reproduced with permission.^[^ ^203^ ^]^ Copyright 2017, Wiley‐VCH GmbH.

Later, a multiplatform acted as both plasmon‐enhanced singlet oxygen (^1^O_2_) generator and photothermal induced drug carrier was developed by He et al. (**Figure** **10**a).^[^ ^202^ ^]^ In their studies, AuNRs with NIR plasmon resonance was first capped by mesoporous TiO_2_ at the end of NRs. The photosensitizer indocyanine green (ICG) was selectively attached to the TiO_2_ caps via copper‐free click reaction. For anticancer drug DOX loading, the side position of the AuNRs was conjugated with CG rich‐DNA strands. By carefully controlling the synthetic process, there were ≈7746 ICG and ≈2278 DOX conjugated to the sides and ends of each TiO_2_ capped AuNRs. Compared with ICG alone, the 2.9‐fold increase in ^1^O_2_ was observed in TiO_2_ capped AuNRs sample. The in vitro experiment further confirmed the killing effect in MDA‐MB‐468 cells (Figure 10b).

In addition, the DNA programmed self‐assembly technique is also a powerful tool for creating light‐responsive assemblies. In this field, Gao et al. developed DNA‐directed chiral shell‐satellite (SS) nanoassemblies with different sizes based on galvanic reaction (Figure 10c).^[^ ^203^ ^]^ In this work, CS AgNP(18 nm)@AuNP (CS5/SS5: 5 nm AuNPs, CS15/SS15: 15 nm AuNPs) structures were first assembled via DNA hybridization. Next, the HAuCl_4_ solution was mixed with CS solution to form the SS NPs based on galvanic replacement chemistry. The structure of the SS was revealed by TEM and EDX‐mapping, which showed that the solid Ag core had been transformed into a hollow Au‐Ag alloy shell. Importantly, the chiral‐optical measurements demonstrated that there was no CD signal from SS5 and CS5, and negative signal for SS15 and CS15 without any cysteine modifications. A symmetrical CD signal at 523 nm was observed when the SS5 and CS5 were modified with D‐/L‐ cysteine, while no detectable CD signal was found from DL‐cysteine modified SS5 and CS5. The results showed that the chiral signal originated from the plasmon‐chiral molecular interactions induced CD response. More detailed characterization revealed that the chirality from SS15 and CS15 was due to the asymmetrical dipole‐dipole interactions. Meanwhile, the chiral SS nanoassemblies could act as photosensitizers to generate ^1^O_2_ under different circular polarized light (CPL) irradiation. For right circular polarized (RCP) irradiation, the SS15 exhibited the highest ^1^O_2_ production and no ^1^O_2_ production after being modified with D‐cysteine and with L‐cysteine, respectively. In particular, the chirality of these assemblies could be maintained after being cultured with HeLa cells. The in vivo experiments showed chiral light‐dependent tumor ablation ability (Figure 10d). Based on DNA hybridization, the same group also developed other types of dynamic heterostructures for bio‐detection and imaging,^[^ ^47^, ^102^, ^103^, ^204^, ^205^, ^206^, ^207^ ^]^ cancer therapy.^[^ ^94^, ^208^ ^]^

Due to the programmable and adjustable features, the DNA origami nanostructures can serve as efficient delivery vehicles for therapeutic drugs, and even the biomolecules via blocking the interaction between the proteins and the environment.^[^ ^13^ ^]^ To this end, Ding and co‐workers developed triangular and tubular DNA origami nanostructures as anticancer drug carriers for DOX‐resistant cancer cell therapy.^[^ ^178^ ^]^ In their studies, the DOX was non‐covalently loaded to the DNA origami nanostructures via intercalation. The DOX‐loaded origami nanostructures demonstrated enhanced drug accumulation and exhibited prominent therapeutic efficacy toward both MCF‐7 cells and drug‐resistant MCF‐7 cells. In their recent studies, the linear tumor therapeutic gene (p53) or linear small hairpin RNA (shRNA) with DOX were co‐loaded into DNA origami nanostructures for synergistic cancer therapy of multidrug‐resistant (MDR) tumors in vivo.^[^ ^209^, ^210^ ^]^ Castro group reported a rod‐like DNA origami nanostructure as the drug carrier for circumvention of daunorubicin resistance in a leukemia cell line model.^[^ ^211^ ^]^ The daunorubicin‐loaded DNA nanostructures were able to enhance drug efficacy in MDR cells. In 2019, Wiraja et al. investigated the transdermal delivery of 20–200 nm framework nucleic acid (FNA) through topical applications.^[^ ^212^ ^]^ They found the size‐dependent skin penetration phenomenon, and 17 nm tetrahedral FNA showed the highest penetration from skin periphery. The structural integrity of the FNA could be maintained during the skin penetration process. The drug loaded FNAs demonstrated effective chemotherapeutic agent delivery and tumor inhibition.

Specific peptide architectures were shown to stabilize efficiently 3D wireframed DNA constructs for a variety of biomedically relevant environments, including magnesium‐ion depletion and the presence of degrading nuclease.^[^ ^30^ ^]^ This study also demonstrates that peptide‐coated DNA constructs provide a controllable of release anticancer drug, as well as can carry desired biomolecules on the DNA surfaces. Thus, a designed DNA scaffolds offer multifunctional and environmentally robust molecular platform for building more complex bio‐structures for nanomedicine and biosensing.

Different from the traditional protein delivery strategies, the DNA origami nanostructures recently were explored as the containers for protein encapsulation.^[^ ^196^, ^198^ ^]^ Kostiainen and co‐workers introduced a virus capsid protein coated DNA origami nanostructure for enhancing cellular delivery.^[^ ^199^ ^]^ In this work, the rectangular DNA origami nanostructure was used as the template for the assembly of cowpea chlorotic mottle virus capsid proteins through electrostatic interactions. After incubation with HEK293 cells, the results showed the proteins coating improved the cellular attachment and demonstrated 13‐fold enhancement in origami delivery compared with bare DNA origami nanostructures. Inspired by the biological systems, Stevens group introduced multiple proteins encapsulated NDA nanoflowers using RCA approach and the applications in intracellular protein delivery.^[^ ^213^ ^]^ In their work, the cytotoxic protein RNase A was successfully delivered to the cells without losing its biological function and structural integrity, thereby inducing significant cytotoxic effects over the free protein.

**Figure 11:**
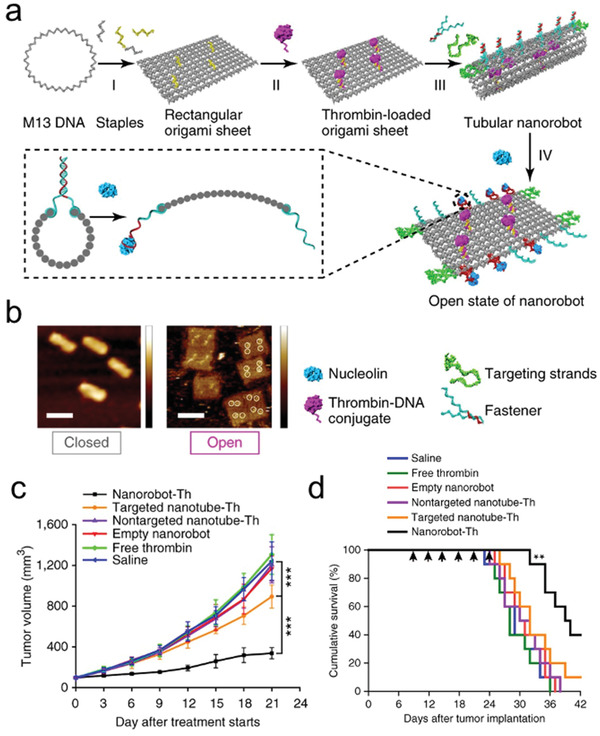
a) Scheme of the fabrication of thrombin‐loaded DNA origami nanorobot and its reconfiguration in response to nucleolin binding. b) AFM images of the DNA origami nanorobot in closed and opened states. c) The relative tumor growth curves of MDA‐MB‐231 tumor‐bearing mice after various treatments, and the corresponding survival curves. Reproduced with permission.^[^ ^198^ ^]^ Copyright 2018, Springer Nature.

Inspired by robotic technology, the Church group developed an autonomous DNA origami nanorobot, which was capable of delivering proteins or NPs to the specific cells.^[^ ^196^ ^]^ Interestingly, the organization fashion of the loaded payloads could be controlled through an aptamer encoded logic gate, responding to different cues. The selective regulation function of protein encapsulated nanorobot was tested with two different types of cell‐signaling stimulation in tissue culture. Inspired by this pioneer work, Zhao and Ding's groups recently reported a thrombin loaded DNA origami nanostructure as a nanorobot for in vivo cancer therapy in response to molecular trigger (**Figure** **11**).^[^ ^198^ ^]^ The rectangular DNA origami nanostructure was used as basis for thrombin loading. The addition of fasteners and targeting strands would induce the self‐assembly of tubular DNA nanorobot which was functionalized on the outside with a nucleolin‐targeting aptamer, a protein specifically expressed on tumor‐associated endothelial cells, and thrombin within the inner cavity. In the presence of nucleolin, the tubular DNA nanorobot would re‐configurate into rectangular DNA nanosheet and expose the loaded thrombin. After injecting the DNA nanorobots intravenously into tumor‐bearing mice, the thrombin was efficiently delivered to the tumor‐associated blood vessels and resulted in intravascular thrombosis. The nanorobot triggered thrombosis would induce the tumor necrosis and tumor inhibition. The nanorobot was proven safe and immunologically inert in mice and Bama miniature pigs. By employing the pH‐responsive i‐motif DNA locking strands in the rectangular DNA nanosheet during its reconfiguration process, Ding and co‐workers reported a rectangular DNA nanosheet‐based nanodevice vaccine for cancer immunotherapy very recently.^[^ ^214^ ^]^ Such stimuli‐responsive systems could open a new avenue for developing personalized cancer vaccines.

In addition to cancer therapy, the DNA‐based nanostructures were also widely explored for the treatment of alleviating acute kidney injury,^[^ ^170^ ^]^ wound healing,^[^ ^215^ ^]^ Parkinson's disease,^[^ ^216^ ^]^ and other diseases.^[^ ^217^, ^218^, ^219^, ^220^ ^]^

## 5 Conclusions and Perspectives

In the past decade or so, a lot of impressive progress has been made with regard to DNA nanostructures and their biomedical applications (**Table** **1**). The unique chemical and programmable features enable us to have a better understanding of their structural properties. Different from other building units (such as peptides and proteins), the DNA strands allow for the bottom‐up self‐assembly of complex nanostructures with controllable morphology and distance by turning the length of DNA strands. Therefore, the DNA‐templated self‐assembly nanomaterials have attracted increasing attention from fundamental design to multifunctional nanodevices. However, the research of DNA programmable nanomaterials involves material science, chemistry, biology, physical and computer science, such field is highly challenging and with ample opportunities.

1) One of the main barriers of the extensive applications of DNA nanostructures is the high cost of the DNA templates and low yield. The more complex a structure is, the more DNA strands are needed. For example, the construction of DNA origami usually needs hundreds of single strands, which restricts their practical applications. This motivates us to develop more effective strategies to obtain the DNA templates. Also, this requires us to simplify the current synthetic protocols to improve the product yields.
2) Up to now, direct conjugation of DNA on colloid nanostructures is limited to very few nanomaterials (such as Au). The Au was the first example that could be directly conjugated with DNA strand, thanks to the strong interaction between the Au and the thiol terminated DNA strands. After that, lots of work has been done in the design of 2D and 3D architectures, especially Au‐based architectures. However, very few other examples have been found due to the weak interaction between the DNA strands and the colloidal nanoparticles. Moreover, several steps are required to conjugate the DNA strands (Pd for example). In this way, the precisely controlled assembly of other metal nanostructures with DNA would be very difficult. Therefore, more effective conjugation strategies are urgently needed for other colloid nanostructure conjugations.
3) Although great efforts have been made in the biomedical applications of DNA‐based materials, long‐term biological stability remains an issue. Different methods have been used to test the stability of DNA‐based materials (e.g., DLS, cytoTEM), while there is little knowledge about the detailed mechanism of the DNA nanostructures in vivo. This emphasizes the importance of more detailed studies of DNA stability both in vitro and in vivo, which requires the collaborative research from different fields.

**Table 1:** Summary of self‐assembled DNA nanostructures

Type	Composition	Binding force	Biomedical application	Advantages	Drawbacks
Single nanoparticle systems	Au	Chemical bonding	Biosensing	High biostability, sensitively	Complicated conjugation procedures
	Rare earth NPs	Chemical bonding	Biosensing, bioimaging	Low background, deep penetration	Easy aggregation
	QDs	Chemical bonding	Biosensing	High sensitively	Low conjugation efficiency
	MOFs	Chemical bonding, encapsulation	Drug delivery, therapy	Controlled drug release, improved cellular uptake	Physical absorption
	2D materials	Noncovalent binding	Biosensing	High sensitively	Low limit of detection
Clusters and extended organization	Un‐, bi‐, ter‐, quater‐NPs	Chemical bonding	Drug delivery, therapy	Multifunctionalities	Low yield, long‐term stability
DNA origami‐templated particle system	DNA origami, NPs, biomolecule	Chemical bonding	Drug delivery, therapy	High delivery efficiency	Costly, long‐term stability

## Conflict of Interest

The authors declare no conflict of interest.
